# Sensing Architecture for Terrestrial Crop Monitoring: Harvesting Data as an Asset

**DOI:** 10.3390/s21093114

**Published:** 2021-04-30

**Authors:** Francisco Rovira-Más, Verónica Saiz-Rubio, Andrés Cuenca-Cuenca

**Affiliations:** Agricultural Robotics Laboratory, Universitat Politècnica de València, 46022 Valencia, Spain; vesairu@upv.es (V.S.-R.); ancuecu1@upv.es (A.C.-C.)

**Keywords:** crop monitoring, agricultural robots, field scouting, sensor architecture, digital farming, proximal sensing, spectral indices, big data, machine learning, recommendation engines

## Abstract

Very often, the root of problems found to produce food sustainably, as well as the origin of many environmental issues, derive from making decisions with unreliable or inexistent data. Data-driven agriculture has emerged as a way to palliate the lack of meaningful information when taking critical steps in the field. However, many decisive parameters still require manual measurements and proximity to the target, which results in the typical undersampling that impedes statistical significance and the application of AI techniques that rely on massive data. To invert this trend, and simultaneously combine crop proximity with massive sampling, a sensing architecture for automating crop scouting from ground vehicles is proposed. At present, there are no clear guidelines of how monitoring vehicles must be configured for optimally tracking crop parameters at high resolution. This paper structures the architecture for such vehicles in four subsystems, examines the most common components for each subsystem, and delves into their interactions for an efficient delivery of high-density field data from initial acquisition to final recommendation. Its main advantages rest on the real time generation of crop maps that blend the global positioning of canopy location, some of their agronomical traits, and the precise monitoring of the ambient conditions surrounding such canopies. As a use case, the envisioned architecture was embodied in an autonomous robot to automatically sort two harvesting zones of a commercial vineyard to produce two wines of dissimilar characteristics. The information contained in the maps delivered by the robot may help growers systematically apply differential harvesting, evidencing the suitability of the proposed architecture for massive monitoring and subsequent data-driven actuation. While many crop parameters still cannot be measured non-invasively, the availability of novel sensors is continually growing; to benefit from them, an efficient and trustable sensing architecture becomes indispensable.

## 1. Introduction

When data are not taken and properly used, resources are often misused. Water is a limited resource whose use in agriculture has to be sustainable. The abuse of fertilizers is threatening the environment at a global scale. Tributaries and streams throughout the Mississippi river basin, for instance, are suffering from an overload of nutrients due to excessive use of fertilizers [1], and, in Valencia (Spain), the water pumped from some irrigation wells has such a high concentration of nitrate that agronomists advise against the use of fertilizers containing nitrogen; by taking soil and water data before fertilizing, soil health deterioration can be avoided in the long run. At present, there is a need for management practices that prioritize long-term sustainability over short-term profit, and, in this context, decisions driven by consistent data coming from proximal sensing may contribute to attain a circular economy if nutrients and fertilizers are applied only as needed [2] in a regenerative agriculture where no waste goes to the soil and aquifers. The reputation of a wine over time relies on the reproducibility of grape properties season after season, which is favored by the systematic acquisition of objective data. For wine makers, the availability of practical tools allowing the methodical recording of field data sets the basis for reaching long-term profit and financial stability over maximum earnings in a single vintage. This financial stability is key for assuring economical sustainability, whereas the systematic monitoring of water status allows the rational use of irrigation water, which leads to environmental sustainability, the two pillars upon which data-driven agriculture is founded.

Despite the multiple benefits brought by data-driven agriculture, its practical implementation has been undermined by the difficulty of getting consistent data at the right periodicity, with the necessary precision, and with a minimum spatial resolution (data points per square meter) to apply statistics, geostatistics, and artificial intelligence techniques. As a result, invasive sampling is still the most extended technique today to extract information about the fundamental parameters involved in crop growth and fruit development. Typical invasive measurements include the hydric status where leaves are removed from the plants and introduced in a pressure chamber; nutritional status where several leaves are removed, packed, and sent to a specialized chemistry lab; and pest infestation counting where traps or leaves are sent to the entomology laboratory for insect counting and identification [3]. For all these examples, the mere choosing of the leaves for canopy sampling is already introducing subjectivity to the measuring process before it starts. In addition, their applicability to monitor fields in a regular basis is unfeasible due to the large number of samples required for a meaningful representation. This fact, unfortunately, prevents the systematic use of these techniques at a large scale for cost-efficiency reasons. Sampling every meter along each row of an orchard, and with a row spacing of 4 m, for instance, would yield 2500 measurements per hectare. If we further assume that each measurement takes 1 min including displacements between adjacent points, and an average labor cost of 15 €/h, every map would cost 625 €/ha. When several monitoring sessions are needed each season, the costs and logistics invested become mostly unfeasible for the average grower.

Unlike invasive measurements, non-invasive techniques allow the retrieval of data without establishing physical contact between the measuring device and the targeted material. In addition to avoid the destruction of leaves or fruits, their main advantage is the speed at which data can be taken, further enhanced by scouting vehicles. However, not all relevant parameters are measurable non-invasively; most berry components, for example, cannot be determined without interfering with the fruits. Non-invasive techniques may be classified as remote sensing techniques mostly related to satellite imagery or unmanned aerial vehicles, and proximal sensing techniques based on either hand-held sensors or onboard sensors that can work at a given separation distance, and therefore enable on-the-fly monitoring. Hand-held sensors have the advantage of allowing a rapid assessment of crop parameters, which is an important step forward with respect to wet chemistry determinations that typically require long processing times. In addition to the high purchasing cost that some of them have, their main disadvantage is the need to be very close to the target, sometimes even touching it, which excludes the possibility of making measurements from moving vehicles without having to stop to approximate the sensor to the target. On top of this, some hand-held devices can weight several kilograms and are physically demanding after hours of monitoring work in the field. Nevertheless, light cost-effective hand-held sensors are resourceful for a quick isolated assessment, but not for systematic monitoring and mapping. Airborne monitoring, on the other hand, has been helpful for staple crops such as corn, wheat, or soybeans, which are typically produced over large extensions where the entire terrain is covered by the crop. Specialty crops, by contrast, pose three fundamental challenges to regular monitoring: first, trees are separated and outlined in rows, leaving large portions of soil uncovered that complicate the treatment of aerial images and the association of data to specific trees [4]. Second, most of the yield is borne inside the canopy or in medium-low heights, being most of the fruits not accessible from zenithal views. Third, very few specialty crops are ready for mechanical harvesting [5], and when available, there are no real-time yield monitors to track the spatial distribution of production, and thus apply precision farming tools. Nevertheless, the arrangement of trees in vertical trellises, such that conventional spherical canopies are conformed into continuous walls that highly reduce fruit occlusion and facilitate the navigation of vehicles, has meant a critical step forward to the applicability of precision agriculture (PA) to orchards. These supporting structures were first implemented in vineyards for wine production but are currently being adopted by other high-value crops such as olive trees, almonds, cherries, and apples [4]. The combination of proximal sensing, ground vehicles, vertical trellises, and massive data is opening a wide offer of opportunities never seen before for digitalizing agriculture [3].

Proximal sensing from ground vehicles facilitates the systematic acquisition of massive field data, especially in the case of specialty crops and orchard production, but, before gathering all these data, it is important to determine what can actually be measured, and its relationship to relevant agronomical parameters that are essential for a sustainable production. The change of light reflectance with plant vigor, water content, and other factors determined by chemical and morphological characteristics of the surface of leaves, leads to the development of vegetation indices as a practical quantification tool for the status of vegetation [6]. Water stress can be assessed by thermal infrared reflectance associated to canopy temperature, which tends to rise when there is a deficiency in water content as a consequence of the closure of leaf stomata to avoid further evapotranspiration [7]. The thermal infrared emission follows the blackbody radiation law, and thus allows its association to leaf temperature, providing an indirect estimation of hydric stress since stomata dynamics regulates transpiration rates [7,8]. Unfortunately, the popular index CWSI (Crop Water Stress Index) poses many difficulties for sensing from a moving vehicle, as locally referenced temperatures for stressed and unstressed leaves are necessary [9]. As a result, water status has mostly been measured from hand-held radiometers [10]. The nutritional status of plants can sometimes be noticed by visual patterns and color changes in the leaves, but these visible changes are typically identified when the imbalance of a specific nutrient has already caused a significant damage to the plant. NDVI (Normalized Difference Vegetation Index) has been the universal index used to monitor nitrogen content in the leaves of staple crops and vigor in vineyards, with both hand-held devices and on-vehicle sensors including aerial platforms. This index is the ratio of the difference between red and NIR reflectance divided by their sum, and can range between −1 and 1, with negative values associated to non-vegetal surfaces such as water, and typical values for vegetation between 0.1 and 0.9, with higher values for thicker canopies. Although vigor and growth are related to the nutritional status, there is no well-established correlation that allows the direct assessment of nitrogen uptake in leaves with the NDVI. In fact, other complementary indices have been proposed to bring some light to the non-invasive estimation of the nutritional state in plants and trees. However, the real time calculation of alternative indices requires spectral profiles at several bands, which is currently attainable with hyperspectral imaging, not quite ready yet for field cost-effective applications. Several such indices have been related to the chlorophyll content in the leaves. In particular, CI (Chlorophyll Index) is strongly linked to leaf chlorophyll concentration [11] and TGI (Triangular Greenness Index) combines the spectral features of chlorophyll related to reflectance at 670, 550, and 480 nm [12]. In general, during fruit maturation processes, the level of pigments and sugars increases while the acidity diminishes. The current approach to track ripening, and therefore determining harvesting readiness, is by sampling in the field and analyzing fruit properties as sugar and acidity with laboratory equipment.

Many growers find dire difficulties to deal with large amounts of data, often packed in unfriendly formats. Even when service companies process data to deliver more elaborated information to end-users, farmers are seldom provided with decision-making information regarding the actual needs of the monitored field. The underlying reason for this roots in the fact that there are multiple and subtle correlations among hydric stress, nutritional status, ripeness, weather conditions, soil properties, and other factors that influence the quantity and quality of yield in short and long terms [6,7,8,9,10,11,12]. As a result, there is a need for moving forward from mere monitoring of crop conditions to decision-making support, and, for such a leap forward, it will be necessary to gather massive amounts of data and implement robust AI-based techniques capable of extracting relevant information for the growers [3]. In other words, what most field managers and fruit growers probably need is a set of recommendation engines capable of digesting complex and massive field data to eventually deliver straightforward operational actions. Algorithms transform data into relevant recommendations by finding, calculating, and ranking the most interesting correlations for users [13]. Regression techniques use data to predict outcomes; in contrast, recommendation algorithms sort outcomes into discrete categories such as classes or zones. Data-driven approaches need both, as the combination of classification with regression leads to predictive personalization [13]. Personalization, indeed, is essential for decision-making in agricultural fields, where site-specific climatic or terrain traits may impact the interpretation of data. In the wine world, for instance, this is particularly known as the terroir.

The majority of recommending algorithms draw their power from the optimal fit between statistics and machine learning (ML). Given that grower knowledge based on field experience is required to train the system in such a complex scenario as crop growth and fruit bearing, supervised machine learning techniques or those based on rewards will provide an initial modeling scenario, but unsupervised learning will be resourceful once this grower knowledge is well coded into accessible parameters, as in the clustering technique described in the results Section. Supervised learning requires an input and an output to learn a mapping from the input to the output; unsupervised learning, on the contrary, has no predefined output and only the input data are available. The aim of the latter is thus finding the regularities in the input, as for instance when clustering to find groupings of input [14]. Unsupervised learning is attractive because, once programmed, it does not require further training. Supervised ML, however, requires deciding the size of the training set, which typically influences results. The need of a vast dataset may threaten the viability of the solution. Fortunately, that is not always the case. In a semi-self-supervised ML system to detect oranges as an intermediate stage for autonomous harvesting, an acceptable solution was reached with only 110 labeled images [15]. At the time of designing a PA-oriented recommendation engine, the first question to address is what type of actuation the algorithm is capable of recommending, out of the many operations related to crop production. The output can be as wide as finding the optimal crop (out of 10) for given soil properties (depth, texture, pH, color, drainage, etc.), a problem faced in India to increase productivity by improving net yield rate [16]. However, a much more common need is to seek advice on regular operations that affect the majority of crops several times per season: irrigation and pest control. The former case has been tackled by a decision support system (DSS) to determine variable rate irrigation in corn [17]. Despite their advantages for sustainability, there has been a limited adoption of variable rate irrigation systems, and, according to these researchers, one potential reason for that is the lack of science-based information. This DSS recommends the amount of water applied by a pivot in four treatments, based upon the dynamic calculation of the crop water stress index (CWSI). In a similar fashion, the latter case was attempted with a DSS designed for pesticide application—dosage and method—to fight sunn pest (*Eurygaster integriceps*) in wheat and barley [18], a knowledge-based system that integrated farmer experience in a logic that considers plant growth stage, plant variety, irrigated or dry farming, damage type (leaf, stem, and ear), and the phenology of sunn pest. These knowledge-based approaches present important advantages to other unsupervised recommendation engines. On the one hand, they can avoid cold start issues, and, on the other hand, they can be easily designed to elude wild recommendations. However, they tend to be data-demanding, and, although currently available field data are, arguably, not close to big-data standards, massive data acquisition is today achievable and prone to feed on-vehicle expert systems and recommendation engines. This article proposes a generic architecture for scouting ground robots capable of registering massive field data, and presents a use-case focused on differential grape harvesting for elaborating two distinct wines from the same vineyard plot.

## 2. Materials and Methods

When measurements can be carried out from a relatively short distance, say up to 5 m, two critical conditions meet: the vicinity of targeted plants or trees may be accurately monitored (plant-specific precision) and continuous measurements from a moving platform (driven or autonomous) can be achieved, moving from undersampling to massive sampling, which eventually might approach big data characteristics. These are the conditions that endow proximal sensing with such a large potential for digital agriculture and data-driven decision-making. Nevertheless, some cautionary reflections are to be pointed out. First, the data acquisition rate may improve in efficiency by increasing the displacement velocity of the supporting platform; however, that will only be true if the sensors onboard can work at higher frequencies. Some commercial devices, such as spectral reflectance sensors, cannot work at sampling rates over 2 Hz, forcing developers to find a compromise between moving velocity and the number of points acquired per hectare. Another important question to deal with is the configuration of a proximal sensing application that can work cost-efficiently for large plots, as ground vehicles usually move slowly, e.g., under 10 km/h. Based on such challenges, and the fact that a decision-making recommender will be as good as the data that feeds it, the following subsections develop the relevant features envisioned for a monitoring terrain vehicle, describing the most popular sensing techniques currently used—or with high potential—for vineyards and groves.

### 2.1. Map Configuration for Agile Data Sharing and Interpretation

Data storage and data processing very often end in oversized tables or matrices. While clearly useful for finding correlations, trends, and outliers, they typically result overwhelming for the average end-user. An advantageous architecture for orchard monitoring must include a corresponding advantageous way of displaying results, and numerical series do not seem to be the right choice. Two-dimensional (2D) zenithal maps of agricultural fields provide an intuitive representation that is easy to understand for most practitioners, where specific areas known by the growers can be easily identified and treated. Nevertheless, for maps to be practical, the information conveyed through them must be unambiguously interpreted, and for that, we need to define the coordinate system, its origin, the map resolution, and the working units. Global positioning with GNSS (Global Navigation Satellite System) receivers grants compatibility among maps and along seasons, but caution must be followed at the time of choosing coordinates. Geodetic coordinates latitude and longitude should not be employed to represent flat fields, despite the fact GNSS receivers use them to output location information. The reason behind is that geodetic coordinates are actually spherical coordinates, and they are not appropriate for calculating distances, angles, and areas contained on a plane; for such a case, the suitable solution is brought by flat coordinates that allow the use of more familiar Euclidean geometry. The most popular flat coordinate systems used in agriculture are UTM (Universal Transverse Mercator) and LTP (Local Tangent Plane). The former uses coordinates X and Y related to a standardized grid, whereas the latter uses east and north related to a user-defined local origin. A local origin defined by each user presents interesting practical benefits: on the one hand, the map will be intuitive for the user who will always know where the origin is (unlike UTM where the origin is typically far away and unknown to the user), and on the other hand, the magnitude of coordinates and distances will be manageable as numbers will be kept small. In addition, the orientation of tree rows and other relevant field features will be facilitated by cardinal directions east (E) and north (N). In light of these advantages, the LTP coordinate system (also called NED) will be the representation system recommended in practice, and thus the one used in this paper. A detailed procedure to transform geodetic coordinates to the LTP coordinate system is explained in [19].

**Figure 1:**
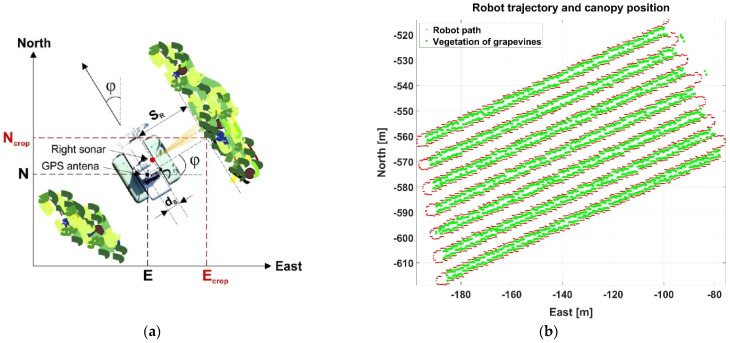
Derivation of crop coordinates from vehicle coordinates: (**a**) problem description; and (**b**) results on a commercial vineyard from a scouting robot.

Precision agriculture relies on global positioning to precisely locate inputs, machines, crops, and yields in any given field. GNSS receivers, mostly GPS, are the universal devices to retrieve geodetic coordinates in real time, with a wide range of designs, accuracy, and cost. However, receivers always output the coordinates of the antenna, and inconsistencies may appear if accuracy is granted for the receiver but the antenna is poorly located with respect to the targeted objective. This is relevant for plant-specific applications, where the position of each plant or tree is what matters, but the antenna may be placed somewhere on the roof of the monitoring vehicle. This is understandable, however, due to the challenges of rendering canopies in real time, as it requires sensing the position of the canopy related to the vehicle in real time, as well as keeping track of the vehicle’s heading. However, when these two parameters are accessible, as explained in Section 2.2, it is possible to plot both, the vehicle’s trajectory and the position of the sensed canopy (Figure 1b). This is even more significant when a monitoring vehicle only senses on one side of the row. Equation (1) specifies the generation of canopy coordinates E_crop_ (m) and N_crop_ (m) for such a case, when the monitoring vehicle only senses the right side of the canopy and the GNSS antenna is located in an arbitrary place within the vehicle. Figure 1a schematizes this scenario where the 2D LTP coordinates of the vehicle (GNSS antenna) E, N (m) are known, the measurement of a right-looking distance sensor (e.g. a sonar) S_R_ (m) is available (Section 2.2.2), the (perpendicular) distance d_S_ (m) between the antenna centerline and the position where the distance sensor has been mounted is fixed, and the heading φ (°) is estimated with reasonable accuracy (Section 2.2.4). Figure 1b plots the results of applying Equation (1) to the trajectory of a scouting vehicle monitoring every two rows. Notice that sensing just on one (right) side of the row and skipping every other row results in an irregular pattern regarding the canopy, as all rows are equally spaced in the field. This effect will be later compensated by quantizing the monitored space into regular square cells forming a normalized grid.

(1) $$\{\begin{array}{c} S_{R}=0\to \;\;\;\;\;\;\;\;\;\;\;\;\;\;\;\;\{\begin{array}{c} E_{\mathrm{crop}}=0\\ N_{\mathrm{crop}}=0 \end{array}\\ S_{R}\neq 0\to \{\begin{array}{c} \begin{array}{c} 90{}^{\circ}\geq \varphi \geq 0{}^{\circ}\to \{\begin{array}{c} \Omega \left(\mathrm{rad}\right)=\varphi \cdot \pi /180\\ E_{\mathrm{crop}}=E+\left(d_{S}+S_{R}\right)\cdot \cos\Omega \\ N_{\mathrm{crop}}=N-\left(d_{S}+S_{R}\right)\cdot \sin\Omega \end{array}\\ 180{}^{\circ}\geq \varphi >90{}^{\circ}\to \{\begin{array}{c} \Omega \left(\mathrm{rad}\right)=\left(180-\varphi \right)\cdot \pi /180\\ E_{\mathrm{crop}}=E-\left(d_{S}+S_{R}\right)\cdot \cos\Omega \\ N_{\mathrm{crop}}=N-\left(d_{S}+S_{R}\right)\cdot \sin\Omega \end{array} \end{array}\\ \begin{array}{c} 270{}^{\circ}\geq \varphi >180{}^{\circ}\to \{\begin{array}{c} \Omega \left(\mathrm{rad}\right)=\left(\varphi -180\right)\cdot \pi /180\\ E_{\mathrm{crop}}=E-\left(d_{S}+S_{R}\right)\cdot \cos\Omega \\ N_{\mathrm{crop}}=N+\left(d_{S}+S_{R}\right)\cdot \sin\Omega \end{array}\\ 360{}^{\circ}\geq \varphi >270{}^{\circ}\to \{\begin{array}{c} \Omega \left(\mathrm{rad}\right)=\left(360-\varphi \right)\cdot \pi /180\\ E_{\mathrm{crop}}=E+\left(d_{S}+S_{R}\right)\cdot \cos\Omega \\ N_{\mathrm{crop}}=N+\left(d_{S}+S_{R}\right)\cdot \sin\Omega \end{array} \end{array} \end{array} \end{array}$$

### 2.2. General Sensing Architecture for Crop Observation and Scouting

Overall, according to their specific purpose, we can sort scouting vehicles for orchards into soil, vegetation, or fruit monitoring vehicles. It would be remarkable to find an all-purpose vehicle, but soil sampling typically requires a stop-and-go approach, whereas canopy measurements benefit from on-motion recording to increase efficiency, and it seems a better approach to attempt one challenge at a time. Nevertheless, as fruit bearing typically occurs in the same physical space as vegetation, mainly for trellis-structured crops, some vegetation sensors can also be used for fruit scanning, even though crop monitoring usually refers to vegetation. This paper focuses on crop monitoring vehicles.

**Figure 2:**
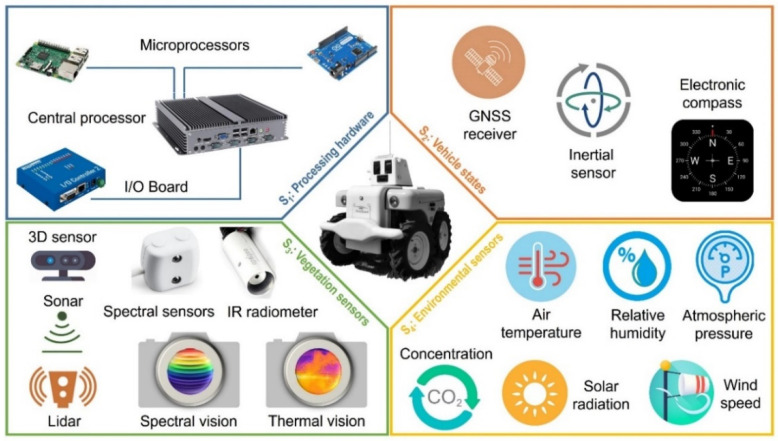
Core systems forming a general architecture for crop monitoring terrestrial vehicles.

Rather than particular difficulties created by individual sensors, the main complexity for crop monitoring vehicles roots in the integration and synchronization of all the sensors with a central processing unit for reliable data gathering. Many times, fortunately, alternative sensors exist for monitoring a determined parameter, and it will be the responsibility of the system integrator to come up with the most stable solution for a given application. The general architecture proposed here for crop monitoring vehicles is structured into four core systems, as graphically illustrated in Figure 2:

- S1-Processing hardware: This system comprises a central processing unit and potential microprocessors associated to specific sensors to speed up or aid in the treatment of their measurements. Associated microprocessors must transfer their results to the central unit at the main cycle frequency. In addition, and due to the complexity posed by the multiplicity and diversity of sensors, a complementary input–output communication board may be connected to the central processing unit. The use-case covered in the Section 3 specifies how a processing hardware system was outlined for monitoring a vineyard.
- S2-Vehicle states: In addition to making reliable measurements, the time-space circumstances around each individual measurement have to be unambiguously determined; without them, monitoring is useless. The most obvious—and critical—piece of information will be the time and global position for each point registered, but the monitoring velocity or the orientation of the vehicle is usually valuable information to know when the vehicle stops, moves in slope, or needs to scan the sunny and shaded side of the canopy separately.
- S3-Vegetation sensors: The goal of this core system is to extract information from vegetation in real time, non-invasively, and from a moving platform. There exist many measuring devices for vegetation, but not all comply with the previous requisites. This system is specific for crop monitoring, and it is the one making these vehicles different from other monitoring solutions. Typical measurements include the physical properties of the canopy, such as volume or porosity, and those based on the spectral response of vegetation, particularly the ones leading to the calculation of standardized vegetation indices.
- S4-Environmental sensors. The interpretation of physiological phenomena in plants and trees often requires complementing vegetation data with environmental conditions, and, preferably, both measurements must occur simultaneously. The particular ambient enveloping each plant has an influence on its response to nutrition, water, growth, evapotranspiration, pest attacks, and so forth. This system represents an advantage to remote sensing monitoring where plant vicinity conditions are out of reach. Usual parameters of interest include air temperature, relative humidity, wind speed, barometric pressure, or carbon dioxide [8,17].

#### 2.2.1. Satellite-Based Positioning

Any crop monitoring vehicle at least needs one GNSS receiver; without it, the maps outlined in Section 2.1 for data sharing and interpretation would not be possible. For the majority of monitoring applications, sub-inch accuracy is not necessary. However, reliability is the key property to pursue, because, once a particular receiver with known accuracy is selected, the goal is to acquire data without interruptions, as mapping has to accidentally cease when global references are no longer available. This is the case, for instance, when canopy monitoring takes place at midday in the summer and temperatures rise over 40 °C; an overheated receiver may stop working and remain unnoticed for a long time while the vehicle keeps moving. To prevent this, receivers should be mounted away from direct sunlight and, when possible, close to ventilated zones. Sometimes it is a good practice to attach a small fan to the GNSS receiver to assure cooling by convection. The position of the antenna is also a vital decision to make. Among GPS error sources, the most difficult to palliate is multipath errors, as receiver errors can be minimized with robustly designed receivers, and atmospheric errors are efficiently compensated by differential GPS (DGPS) solutions. Even though most farmers are not willing to pay a subscription fee for differential signals, wide range public augmentation networks, such as WAAS (USA), EGNOS (Europe), or MSAS (Japan), offer a reasonable trade-off. Multipath errors, unfortunately, depend on each vehicle surroundings, and therefore are unpredictable. Nearby rural buildings or tall trees typically pose a barrier to the straight beams coming from the satellites to the vehicle’s antenna. As a result, an advantageous position of the antenna can palliate the incidence of multipath errors. The rule of thumb would be to locate the antenna at least as high as the average tree height of the orchard being monitored, and even a little bit higher whenever possible. Affixing the antenna to an extendable pole is a good way to adapt the vehicle to the specific needs of each field. The vehicle shown in Section 3, for example, features a foldable antenna that can reach 1.8 m over ground, being sufficient to monitor common vineyards.

Once the GNSS hardware (receiver and antenna) is selected and in place, attention turns to integration, communication, messaging, and software. The communication between the receiver and the central processing unit must be permanently granted. It has typically followed a serial protocol. Robust connectors, such as DB9 for RS-232 protocol or the connectors used with the CAN bus protocol, are preferable to weaker joints such as USB. An important parameter to set when integrating a GNSS receiver is frequency or sampling rate, as it depends on the moving velocity. Basic 1 Hz rates are usually too low, being 5 Hz a good compromise for regular traveling speeds in the orchard. Larger rates may overload the data files with repeated information, as most radiometers and spectral reflectance sensors cannot reach such frequencies. As important as the physical integration of a GNSS receiver is its software integration, and that requires selecting the messages being processed and applying filters to avoid dangerous outliers. As mentioned above, reliability and consistency are the top specifications when selecting receivers, as they bring confidence to the results conveyed through field maps. The vehicle states system (S2) normally requires the acquisition of data captured in two NMEA (National Marine Electronics Association) standard strings: GGA for time, position, and consistency (number of satellites, HDOP, and fix), and VTG for ground speed and heading. These NMEA strings must be read from the receiver at the selected frequency and checked for consistency, parsing only consistent messages and discarding unrealistic estimates. When GNSS incoming data are classified as unreliable, the map-building algorithm should neglect them, being always better to leave field gaps without data than adding corrupted information. Given that onboard mapping requires that geodetic coordinates are transformed to LTP flat coordinates in real time, the integration code should ease the selection of the user-chosen origin of coordinates. However, once fixed for a given field, the origin of coordinates should be kept for the rest of maps in current and future seasons.

#### 2.2.2. Vehicle-Fixed Canopy Location: Sonar and Lidar

**Figure 3:**
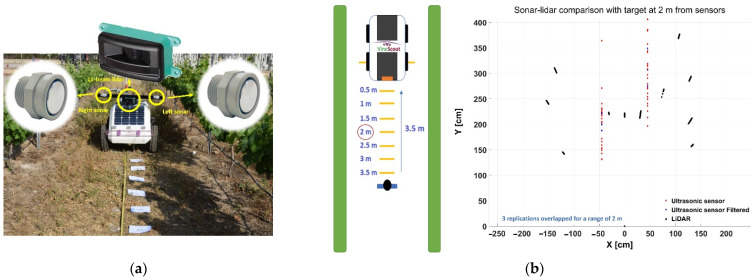
Comparison between ultrasonic and lidar rangefinders in relevant environments: (**a**) experimental setup; and (**b**) results.

Figure 1 evidences the advantages of knowing the precise location of vegetation and Equation (1) shows the need of estimating the distance between the GNSS antenna and the canopy. This distance becomes available when the distance (S_R_) between the monitoring vehicle and the targeted canopy can be measured in real time. Among the diverse sensors capable of estimating distances, ultrasonic sensors (or sonar sensors) and lidar rangefinders present significant advantages for agriculture: their range scope is favorable to proximal sensing, usually up to 5–8 m; cost is, generally speaking, moderate in comparison to other sensing technologies, say up to $1500 for one lidar or three ultrasonic devices; and environmental protection rates of at least IP-65 grant long-term performance in outdoors conditions, which is essential for crop monitoring. Each technology presents different advantages. Ultrasonic sensors are less costly, but as the sound wave propagates in a cone, a network of various devices is needed to cover a vegetation region when various measuring points are necessary. Lidar rangefinders, by contrast, output several measurements at a time and with higher accuracy than sonar. The scouting robot in Section 3 features an ultrasonic sensor facing right side rows to apply Equation (1). For this situation (Figure 1a), one distance (S_R_) at a time is sufficient and the sonar sensor can be a good match, but when several distances have to be acquired simultaneously, a deeper analysis is recommended. Figure 3 shows the comparison of ultrasonic and lidar sensors in field conditions [20]. Figure 3a outlines the experimental setup, where two ultrasonic sensors separated 0.8 m and an 11-ray lidar placed between them tried to detect a person located 2 m ahead, just in front of the lidar head. Figure 3b depicts the results, which yielded very stable readings for the lidar (small black clusters, the three in the middle representing the standing person) and more dispersion for the sonars. The unfiltered sonar readings (red dots) were very noisy, and, although the filtered signal decreased dispersion (blue dots), accuracy was always inferior to that registered with the lidar. Nevertheless, as sonar and lidar are based on different principles, they can bring complementarity to the solution in terms of redundancy; the reflective properties of leaves influence the response of sonar sensors, but canopy gaps may let the narrow beams of lidar pass through without hitting any leaf.

#### 2.2.3. Quantification of Canopy Volume

**Figure 4:**
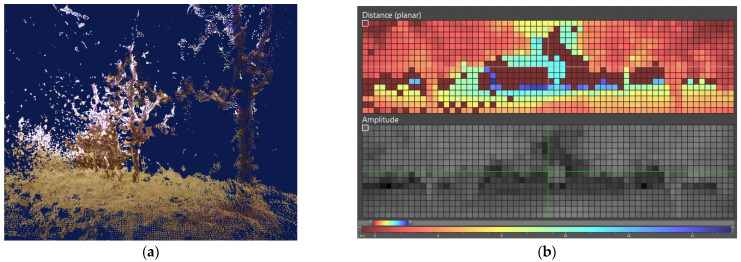
Canopy volume estimation from 3D point clouds: (**a**) stereoscopic vision; and (**b**) time of flight sensors.

A real-time assessment of canopy volume allows the estimation of growth and vigor, which is important for some crops such as grapevines, as well as for the variable-rate application of products, especially fertilizers and chemicals for pest and disease control. The detection of canopy gaps or high variability in tree size within a field plot may result in important product saving, and also in significant environmental benefits. There exist several devices and techniques capable of quantifying canopy volume [21]. For agricultural applications and environments, this measurement has been lately pursued using two approaches: (1) integrating consecutive 2D canopy profiles to obtain the 3D model of vegetation and thus estimate its volume; and (2) directly calculating the volume of a 3D point cloud rendering tree vegetation in real time. The first alternative, based on a 2D profile of canopy, has been attempted with a network of ultrasonic sensors or with a lidar rangefinder (Section 2.2.2). The second approach, which involves acquiring the 3D representation of reality in real time from a point cloud, can be embodied with stereoscopic cameras or with time of flight (TOF) sensors. Stereoscopic vision [22] typically produces higher resolution point clouds than TOF sensors, but the latter can be operated in the absence of light because they include their own illumination source. In terms of robustness, stereoscopic cameras tend to feature lower environmental protection rates. For example, the image in Figure 4b shows the range image of an olive grove taken with a TOF sensor of resolution 16 × 64 and rated IP-67. Figure 4a, by contrast, depicts a 3D point cloud of trees taken with a binocular stereoscopic camera of resolution 400 × 300 and no IP rating.

#### 2.2.4. Row Orientation and Plant-Specific Precision

Section 2.1 exposes the practical advantages of knowing the actual position of plants and canopies rather than a vehicle’s trajectory, mainly in the context of plant-specific precision farming. To do so, however, Equation (1) shows that tracking vegetation requires sensing the distance of the vehicle to targeted vegetation and the vehicle’s heading φ (orientation related to the north) in real time. The former can be straightforwardly attained with sonar or lidar, as explained in Section 2.2.2, but the heading entails certain subtleties that need further attention. In addition, heading information is also useful to determine what side of the row is being monitored, which in turns allows knowing if the monitored side of the canopy was sunlit or shaded. To acquire heading in real time, there are two common approaches: (1) GNSS information from either one or two receivers; and (2) electronic compasses integrating magnetic and inertial technology.

**Figure 5:**
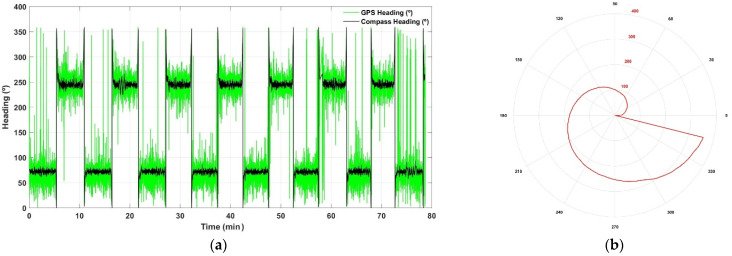
Heading estimation in agricultural vehicles: (**a**) comparison between a GPS receiver and an electronic compass; and (**b**) calibration map for electronic compasses.

The majority of agricultural vehicles today incorporate a GNSS receiver among their regular equipment. However, the coupling of two receivers with the goal of tracking vehicle heading is fairly uncommon, as it implies duplicating cost and complexity for a parameter not always justified. What is more common, however, is to extract heading from the VTG string of a single receiver. This is straightforward and carries no extra cost, but the measurements are so noisy that they practically become useless when an accuracy of a few degrees is pursued. It is not a matter of the proper initialization over the first meters, because large errors remain along entire rows. Figure 5a illustrates this phenomenon for several rows with a length longer than 100 m, when the robot of Section 3 followed a regular pattern along parallel rows. The plot in Figure 5a compares the heading values read directly from the VTG NMEA string of a GPS receiver previously validated in the field [23], with the heading measured in real time with an electronic compass fixed to the vehicle, whose outputs were easily checked, as the actual orientation of the rows is known and can be simply calculated from satellite images. Nevertheless, using an electronic compass is not as easy as setting up plug-and-play devices; it requires an in situ calibration given that electromagnetic interferences within the vehicle will alter its readings. Iron parts, batteries, antenna-supporting magnets, electric motors, cables, and computers will typically have an influence in the magnetic fields around the vehicle. Given that isolating the compass is usually not practical, a compass re-mapping must be conducted as accurately as possible before its extensive use. Fortunately, once mounted in its definite place with the proper orientation and carefully calibrated, it will output stable measurements unless field conditions are heavily adverse, as for example when using metallic posts to guide wires in narrowly spaced vineyards. Figure 5b reproduces the map used to calibrate the electronic compass mounted on the vehicle in Section 3. In practical terms, a calibration map is a table that establishes a correspondence between the reading giving by the electronic compass and the actual heading determined with an external compass not affected by electronic components. Figure 5b plots the actual heading in red and the on-board compass readings in the black polar axes. Notice the non-linearity of the map, as the uncalibrated compass yielded 90° when the heading was actually 101°; both coincide at 180°; 270° is in reality an angle of 254°; and, most importantly, an angle of 346° corresponds to 1°, being the rest of angles up to 360° output by the compass the beginning of the real heading circle from 1° to 24°. Look-up tables are an agile way to implement the calibration map of a compass.

#### 2.2.5. Spectral Indices

The electromagnetic radiation reflected by canopy vegetation and fruits can provide valuable information on the physiological status of plants and trees when specific spectra are isolated and mathematically compared through indices. The common source of incident radiation is the sunlight, which facilitates data acquisition but impedes night monitoring. Different physiological properties require applying different indices [6,7,8,9,10,11,12], and the challenge from a sensing standpoint is to capture and analyze in real time the spectral bands that each index requires. To do so, three different technologies are currently in use: (1) multispectral cameras, which have between two and five equally-sized imaging sensors that capture the same scene at different bands delimited by optical filters; (2) hyperspectral cameras, which scan many lines of narrow spectral width throughout the entire UV–IR range; and (3) compact spectral reflectance sensors (SRS) that directly compute the indices for a given area of interest determined by the sensor field of view and its distance to the target. Each approach has its advantages and drawbacks. The use of hyperspectral cameras in the field is not extended because they are expensive (typical cameras may reach $40,000), computationally intensive, and typically not prepared to stand the harshness of environmental conditions and off-road vehicle dynamics. As a result, they are mostly used for research. Multispectral cameras offer a more robust solution where the high resolution of images allow for accurate pixel-to-pixel calculation of indices. However, cost increases rapidly with the number of imagers with most prices in the range $5000–12,000, and the flexibility of estimating various indices with the same device requires finding the right combination of filters and imagers. For remote sensing applications that involve high-resolution, high-quality images with less cost restrictions, multispectral cameras have resulted a good option. However, ground-based proximal monitoring largely benefits from compact SRS, even if different indices require independent devices. Compact SRS are ruggedized and designed to stand agricultural environments, supply reliable readings with minimum processing needs, and cover well the canopy sections typically monitored in proximity, making them the ideal solution for crop monitoring with terrestrial vehicles. In addition, some SRS sensors include real-time ambient light corrections, which makes them even more accurate and versatile. The vehicle in Section 3, for example, carries two compact SRS sensors, one for measuring NDVI and the other for PRI (Photochemical Reflectance Index), both including an environment-correcting unit and below $1000.

#### 2.2.6. Canopy Temperature

**Figure 6:**
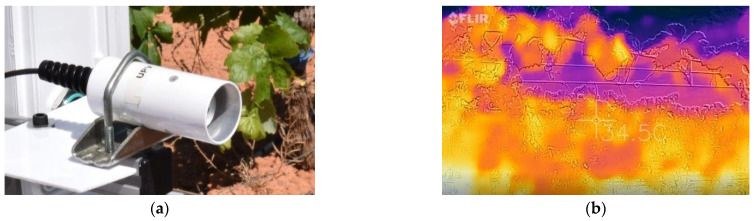
Non-invasive monitoring of canopy temperature: (**a**) infrared radiometer (Apogee Instruments, Inc., Logan, UT, USA); and (**b**) thermographic images of a vineyard from a terrestrial vehicle (FLIR Systems, Inc., Wilsonville, OR, USA).

Canopy temperature has been related, together with other variables, to the hydric demands of water by plants due to the effects of stomata conductance dynamics on leaf temperature [7,8,9]. It is, therefore, an important crop parameter to track. Two alternative approaches have been proposed for the non-invasive measurement of canopy temperature: (1) infrared radiometers that estimate the average temperature of the leaves within a given spot determined by the sensor field of view and its distance to the target, similar to spectral reflectance sensors; and (2) thermographic cameras providing thermic digital images, where each pixel represents a temperature reading of the targeted scene. The field of view of thermographic cameras can be modified by selecting the most appropriate lens for a given application. Likewise, the size of the sensing imager will determine the resolution of the image that best fits a given situation. However, thermographic cameras are, on average, an order of magnitude above infrared radiometers in acquisition cost, and require deeper (image) analyses to calculate temperatures than the simple reading of the average temperature output by radiometers. Some IR radiometers additionally provide the temperature of the sensor. As found with multispectral cameras, for remote sensing applications that require high-resolution images covering large areas, thermographic cameras have been well accepted, but for proximal sensing, where the focusing spot basically covers most of the canopy’s productive area, IR radiometers offer an excellent trade-off. Most of these compact sensors have been built for outdoors, and thus withstand hard environmental conditions while providing stable and reliable readings. The IR radiometer in Figure 6a, for instance, includes an IP-68 marine-grade stainless-steel cable connector and a 18° half-angle field of view. Figure 6b provides a thermographic image of a vineyard row captured from a moving ground vehicle. Notice that calculating the average temperature of the canopy from the thermal image would require first eliminating background pixels related to the sky or supporting structures, and then averaging pixels pointing at vegetation after outlier removal. These operations are complex when executed in real time as they become computationally expensive and prone to error.

#### 2.2.7. Tracking Ambient Conditions at Sub-Meter Proximity

The use case described in the Results Section demonstrates the practical impact of knowing the precise ambient conditions in the close surroundings of every plant, something that is out of reach for remote sensing applications. The idea is keeping a rigorous record of the atmosphere enveloping vegetation as it interacts and influences its growth and physiological response. There exists a wide variety of commercial sensors that facilitate the measurement of climatic and ambient parameters. Reliability and robustness are the main features for selecting these sensors, as integration and sample rate are generally advantageous, taking into account that little changes occur in the atmosphere in less than 1 s, which implies a sampling rate of 1 Hz. A sensing probe is usually a convenient accessory, as it assures that the measuring point in the monitoring vehicle is optimally located in relation to the vegetation. The most important ambient parameters to track are air temperature and relative humidity, which can be complemented with barometric pressure, wind speed, sun radiation, and CO_2_ concentration (Figure 2).

#### 2.2.8. Redundancy and Architectural Choices to Enhance Reliability

According to failure theory, the reliability of parallel systems is always higher than the highest reliability among their individual components. This principle is applied in practice with redundancy and results in duplicating sensors and components that measure crucial variables, such as altimeters in airplanes and obstacle detection systems in auto-steered vehicles. For the case of monitoring vehicles, although redundancy is feasible from the technical standpoint, it is often absent due to practical or economic reasons. This is the case, for example, of vehicle heading (Section 2.2.4). Figure 5a clearly evidences that, even though GPS heading is available, the signal is so unstable that it cannot be used in practice. If the parameter heading is deemed a priority and thus redundancy becomes necessary, other sources than VTG messages will have to be found. A different situation is brought by redundancy in vegetative indices. It is possible to estimate CWSI, NDVI, PRI, and other indices by both compact sensors that provide an average estimate within an area and cameras (multispectral, hyperspectral, and thermographic) that provide multiple measurements through the pixels of digital images. In this case, however, both estimates may have similar reliability and accuracy, but complexity and cost are quite different. IR radiometers and SRS devices do not require elaborate calculations and are very robust, whereas image processing in real time is both computationally and financially intensive. Not being crucial parameters involved in safety or affecting the entire map consistency, keeping just one type of sensors seems reasonable for most ground-based monitoring applications, and, of the two options, compact sensors are probably the best choice.

When a multiplicity of sensors is integrated in a vehicle, choosing the frequency at which the data map is populated becomes an important decision to make. Some sensors, such as SRS and infrared radiometers, require certain time to process the reflected signal, e.g., 0.6 Hz; other sensors are relatively quick, such as the electronic compass that refreshes data at 50 Hz; and the GNSS receiver itself typically works at either 5 or 10 Hz. The question is: What would be an effective trade-off? A high frequency would record many repeated measurements, but a too low sampling rate would miss potential peaks of the signal. The answer rests on the detailed analysis of each particular application. For example, for the vineyard monitoring vehicle described in the use case of the Results Section, it is clear that the low threshold is set by the compact sensors working at 0.6 Hz. Therefore, the lowest sample rate being considered is 1 Hz, i.e., one measurement per second. Ambient conditions—air temperature, relative humidity, barometric pressure, and CO_2_—are not expected to change much every second. However, if the vehicle moves at 2 km/h (0.55 ms^−1^), it covers half meter per second. As it is common to skip GNSS strings occasionally, every missing string would result in at least 1 m without data recording. To compensate for this, the solution envisioned is to populate the map at the GPS frequency of 5 Hz, such that there are at least two or three valid measurements per second and canopies are monitored with data at intervals ranging between 10 and 30 cm.

## 3. Results

This section describes the results of applying the sensing architecture proposed above to a use case: canopy monitoring in vineyards with a terrestrial robot. A commercial vineyard located in Junqueira, Portugal, was continuously monitored between 2017 and 2020 under the EU-funded research project VineScout. The vineyard belongs to the winery Symington Family Estates and has historically produced two distinct wines from the monitored plot. The main sorting parameter has been the degree of hydric status stood by the grapevines, being plants under higher stress levels the ones producing the higher quality wine or, more appropriately, the most-valued wine by customers. The goal of this use case is to determine how the monitored area can be systematically divided into two distinct harvesting zones according to high-resolution crop data. As expected, there are various approaches to sort the field out, from the many-season experience by field managers and oenologist, to NDVI aerial images captured by a drone in a yearly basis, to a complete multivariate analysis processing data from many maps recorded along each season and at different time of the day [24]. In this particular case, to keep the example within a reasonable extension for the scope of this study, we focus on two parameters that have been traditionally related to the hydric state of grapevines: (1) the estimation of vigor from vegetation index NDVI; and (2) the difference between canopy and air temperature (∆T).

**Figure 7:**
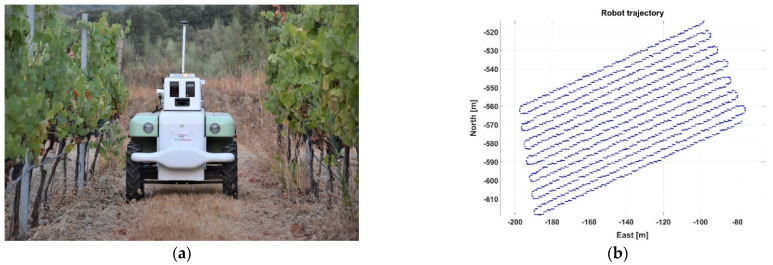
Acquisition of massive data from a vineyard: (**a**) autonomous robot for map generation in season 2020; and (**b**) trajectory traced by the monitoring robot in Map C (Table 1).

In order for the computer recommender to divide the vineyard plot into two distinct harvesting zones, and according to the approach proposed, massive data are needed as the source of knowledge for decision making. The acquisition of proximal sensing data was automated by means of the VineScout robot, whose last version (VS-3) is portrayed in Figure 7a. The trajectory followed by the robot in the generation of Map C (Table 1) is depicted in Figure 7b. The robot is a four-wheel-drive two-wheel-steer platform of 200 kg mass. It is powered by three Li-ion batteries and monitors the right canopy side every two rows. Autonomous navigation requires vertical trellises as crop supporting structures, row spacing in the range 1.8–3 m, and a headland clearance of 5 m. Mild slopes up to 12° and weeds or cover crops below 50 cm are traversable. Canopy temperature was registered by an infrared radiometer (SI-121, Apogee Instruments, Inc., Logan, UT, USA), whereas air temperature was measured with two different sensors: weather sensor BME 280 (Bosch Sensortec GmbH, Reutlingen, Germany) in season 2019 and the ambient sensor T7311-2 (Comet System, s.r.o., Rožnov pod Radhoštěm, Czech Republic) for 2020 season. NDVI was registered with a spectral reflectance sensor (SRS, Meter Group Inc., Pullman, WA, USA), and the distance to the canopy (S_R_ in Equation (1)) was sensed with a ruggedized ultrasonic sensor (UC2000 30GM IUR2 V15, Pepperl + Fuchs, Mannheim, Germany). The SRS has an accuracy of 10% or better for spectral irradiance and radiance values, and a measurement time below 600 ms. The robot is equipped with an electronic compass (SEC385, Bewis Sensing Technology LLC, Wuxi, China) to track instantaneous heading. As for the processing hardware system (S1), the robot comprises a central computer (Irontech, Bescanó, Spain), an input–output connectivity board (NI USB-6216, National Instruments, Austin, TX, USA), and three microprocessors (Arduino, Somerville, MA, USA).

As the differentiating phenomenon to determine zoning is the hydric state stood by the vines, the most significant tests are those taking place during midday or in the afternoon, since water stress conditions were more apparent. Three mapping sessions are analyzed as data source for harvesting zoning, as specified by Table 1. Based on the idea that the force of this (data-driven) approach rests on the amount of data collected in the field, being data consistency taken for granted, we can define the term data pressure (DP) as an analogy from the physical sciences where, in this case, it refers to the number of data points per area unit (points/m^2^). For the tests listed in Table 1, each data point represents a vector including the following measurements: acquisition time, geodetic coordinates, GPS consistency indicators (fix, number of satellites, HDOP), canopy temperature, NDVI, and ambient variables (air temperature, barometric pressure, and relative humidity).

**Figure 8:**
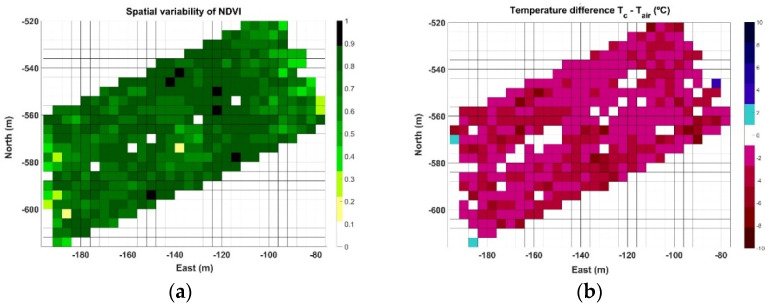
Grid maps for Map A field data: (**a**) spatial distribution of NDVI; and (**b**) ∆T (°C).

The two parameters selected to characterize two different zones of vines are the spectral index NDVI and the difference between canopy and air temperature (∆T). Beginning with Map A, Table 1 indicates that there are 20,124 points registered within approximately one half hectare, leading to a data pressure of 3.7 points/m^2^. In this use case, each point carries information about both parameters (NDVI and ∆T). Each time a map is constructed, each row is characterized by a different number of points, being impossible to make measurements in the same spots when the vehicle traverses the same row in different sessions. This setback, however, is easily circumvented by adopting a grid approach, where each cell represents the averaged value of each parameter within a field portion of expected homogeneity, for example 4 m × 4 m in this use case, considering that row spacing in the vineyard is 2 m and the robot traverses every other row. The NDVI grid map corresponding to Map A is plotted in Figure 8a, and the grid for ∆T is depicted in Figure 8b. As shown in the figure, with a cell size of 16 m^2^, the resolution of the grid is 31 cells in the horizontal dimension and 25 cells in the vertical dimension, summing up to 775 cells in the grid. Interestingly, because the rows are aligned in an orientation southwest–northeast (Figure 7b), the NW and SE corners of the grid are empty, resulting in 400 active cells out of 775, and therefore about 50 points per cell on average. Even though Figure 8a contains the typical dispersion found in agriculture, it is possible to distinguish a strip of higher vigor in direction NW→SE between east coordinates −130 and −100 m. This vigorous strip also has a small ∆T in Figure 8b, about −2 °C, being high ∆T concentrated on east and west field boundaries and more extensively over the west side of the vineyard plot.

**Figure 9:**
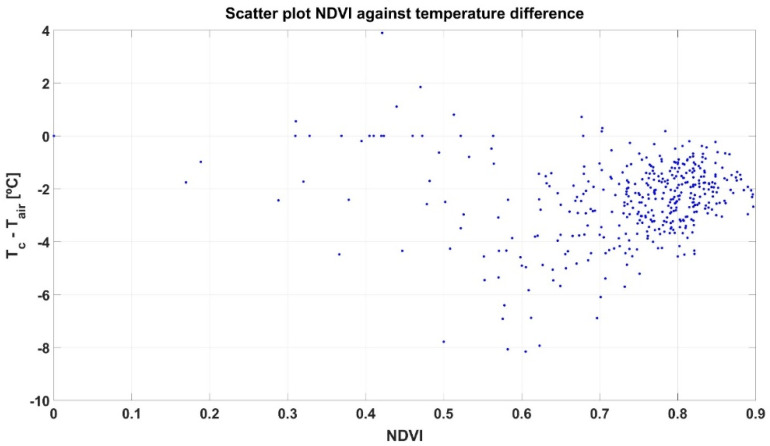
Scatter plot of grid cells for correlation between NDVI and ∆T in the data from Map A.

While the two grids in Figure 8 are quite visual and highlight spatial differences, we should relate them to reach deeper insights before attempting to cluster cells into harvesting zones. Figure 9 shows a scatter plot that merges the information contained in both grids, where the abscissa axis represents the NDVI calculated for each cell, and the ordinate axis shows the temperature difference ∆T. The plot reveals a high concentration of cells agglutinated around NDVI ≈ 0.8 and ∆T ≈ −3 °C.

**Figure 10:**
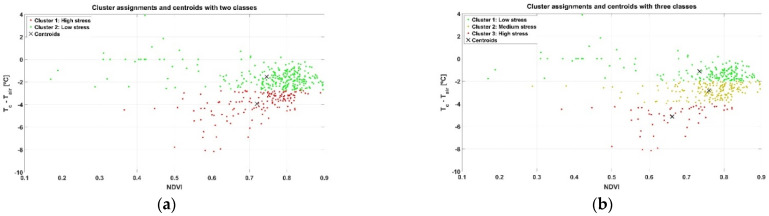
Clustering results and centroids for Map A: (**a**) two classes (k = 2); and (**b**) three classes (k = 3).

**Figure 11:**
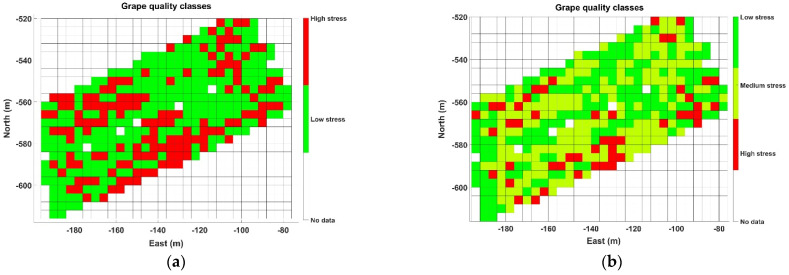
Map A harvesting zones output by the k-means algorithm: (**a**) two zones; and (**b**) three zones.

At first sight, and just following visual inspection, it is difficult to extract conclusions from the plot in Figure 9 regarding zoning. To automate the process and avoid any preconceived bias, an unsupervised learning technique for clustering such as the k-means algorithm was applied, specifically set to use Euclidean distances and execute 1000 iterations. The k-means algorithm requires choosing the number of classes beforehand. Even though only two (wine) classes are possible from a practical standpoint in the vineyard monitored, two potential approaches are initially considered for Map A: (1) straightforward selection of two classes, one representing low stress and the other high stress; and (2) a more restrictive condition for high quality wine, by which three classes of hydric stress are considered (low, medium, and high), merging low and medium stress for one type of wine and leaving high stress cells for the other. Figure 10 plots the resulting clusters and their centroids for both approaches k = 2 (Figure 10a) and k = 3 (Figure 10b), while Figure 11 shows the harvesting zones that result from applying the classes determined by the k-means algorithm (Figure 10) to the actual field plot as monitored in June 2019. Notice that the clustering algorithm gives more weight to the temperature difference than to NDVI at the time of separating classes, something that was not intuitive from the results in Figure 9. Nevertheless, the highest stress is related to lower vigor (smaller NDVI) for both approaches based on the centroids of the clusters.

**Figure 12:**
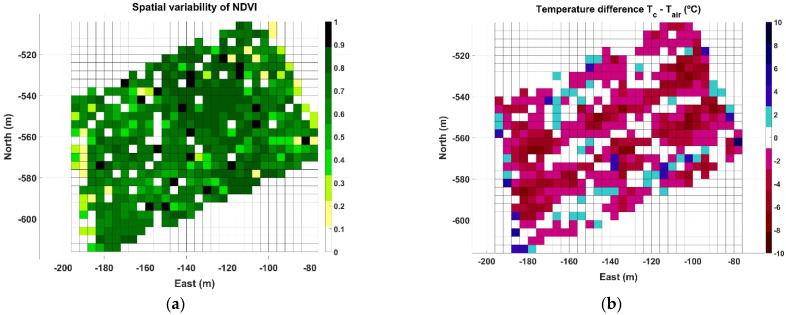
Grid maps for Map B: (**a**) spatial distribution of NDVI; and (**b**) ∆T (°C).

Map B represents the most critical conditions of the three data acquisition sessions, as data were taken in July 2019 during a heat wave with an average ambient temperature for the duration of the test of 40.4 °C, and maximum peaks over 43 °C. Figure 12 illustrates the NDVI grid map (Figure 12a) and the temperature difference ∆T grid map (Figure 12b) for the 0.8 ha monitored in Map B. The spatial distribution of NDVI (Figure 12a), which has been related to vine vigor and growth, continues highlighting the high vigor strip already detected in Figure 8a. The ∆T map in Figure 12b, on the contrary, shows a different pattern where cells representing large negative differentials concentrate along certain rows, enhancing a linear pattern with the same orientation of the vine rows traced by the vehicle path in Figure 7b.

**Figure 13:**
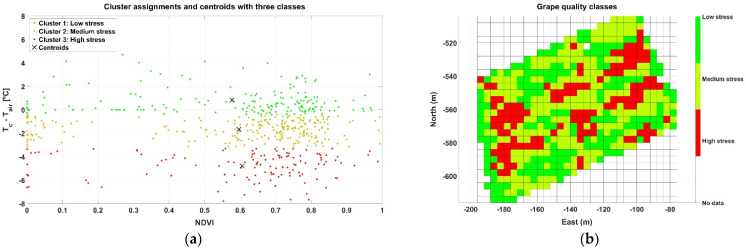
Clustering results and grid for Map B: (**a**) three classes (k = 3) clustering results; and (**b**) zoning.

The zone map in Figure 11a offers a balanced distribution of cells for k = 2 (two zones). However, being demanding in terms of highly distinctive wine traits implies considering only those zones where there is a high likelihood of finding properly control-stressed vines. As a result, the subsequent analysis systematically considers three classes when clustering (k = 3), and therefore three harvesting zones, with the purpose of isolating highly stressed vines and combining low-medium stress in another zone. Figure 13a provides the results of the k-means clustering with its found centroids, while Figure 13b plots the resulting three zones in grid format.

**Figure 14:**
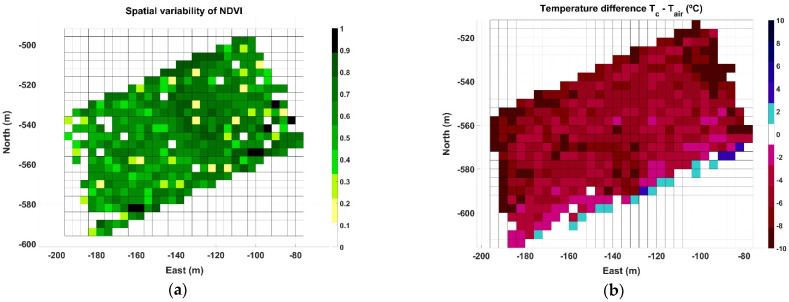
Grids for Map C data: (**a**) spatial distribution of NDVI; and (**b**) ∆T (°C).

The last case studied, Map C, was registered in September 2020, with an average air temperature during the test of 38.9 °C and a range of measurements between 34.2 and 41.2 °C. The average relative humidity was 14%. The grid representing NDVI (Figure 14a) keeps the consistency of the high vigor strip outlined in the previous maps, but the identification of trends in the ∆T grid (Figure 14b) becomes more complex for the cells away from the boundaries.

**Figure 15:**
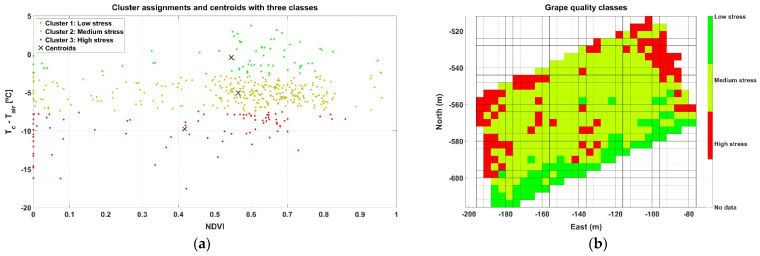
Clustering results and grid for Map C: (**a**) three classes (k = 3) clustering results; and (**b**) zoning.

Despite the lack of clear trends in the spatial distribution of crop parameters, ∆T in particular, unsupervised clustering helps to classify cell properties objectively and unambiguously by means of numerical outputs. High stress for Map C, for example, is characterized by a cluster with a centroid located at NDVI ≈ 0.42 and ∆T ≈ −10 °C, as graphed in the scatter plot of Figure 15a. When grouped cells were spatially distributed in the grid in Figure 15b, systematic zoning showed fewer cells with a high level of stress than in Map B (Figure 13b), coming back, to some extent, to the results of Map A (Figure 11b) collected more than a year earlier.

## 4. Discussion

**Figure 16:**
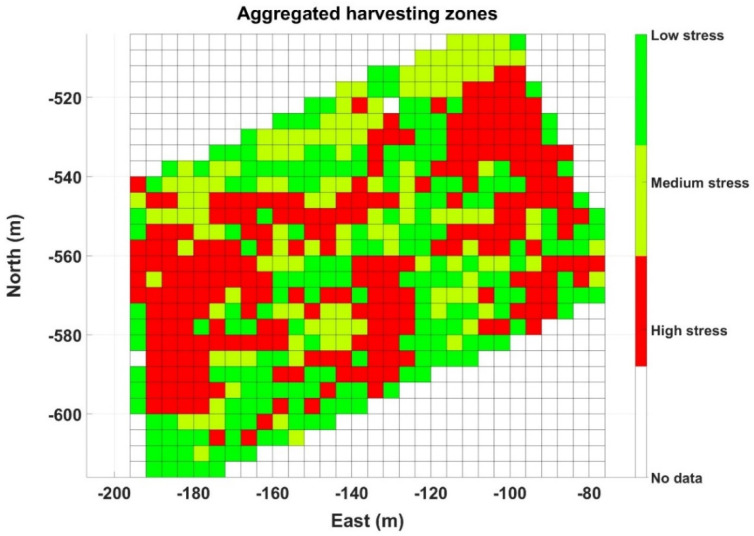
Aggregated map of harvesting zones integrating data from seasons 2019 and 2020.

The three NDVI grid maps estimating vigor for Maps A (Figure 8a), B (Figure 12a), and C (Figure 14a) are consistent in outlining a strip of higher vigor, which also coincides with a high ∆T, either positive or small negative. This, a priori and in advance of a deeper analysis, already reveals signs of an area of lower hydric stress, and therefore distinct properties expected in the grapes. The concluding maps at the time of making a decision, however, are those featuring stress zones (Figure 11b, Figure 13b and Figure 15b). The three maps contain zones that are consistently labeled as high stress, mainly in Maps A and C. However, Map B in particular, the one with the hardest ambient conditions, also highlights specific rows from headland to headland. To ease interpretation, the three zoning maps can be packed up into one, such that every cell retains the highest stress value out of the three final maps (A–C) if it is labeled as high stress at least in one of them. Otherwise, if the cells are labeled as low or medium stress, the final value assigned is low stress, with the purpose of reducing the population of medium stress cells, and thus decreasing map variability to facilitate the sorting of cells into two operative classes. Figure 16 depicts such an aggregated map.

**Figure 17:**
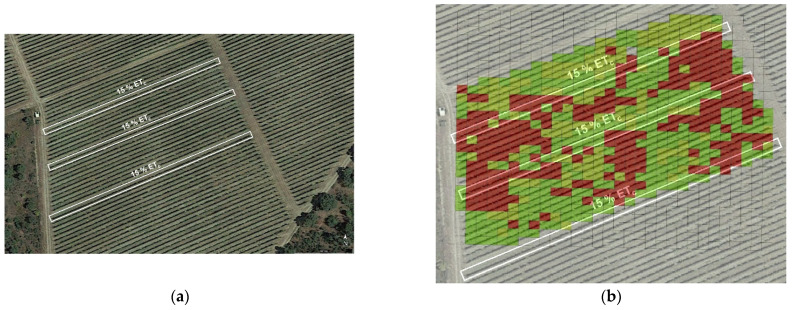
Monitored vineyard: (**a**) satellite image with deficit irrigation lines; and (**b**) potential correlation between low-irrigated rows and high stress deduced from the aggregated zoning map in Figure 16.

In the process of implementing decision support mechanisms based on data, after setting an advantageous sensing architecture, choosing the proper field parameters, gathering massive field data, and using learning techniques to assist in data analysis, we reached the final stage in which a recommendation must be delivered to the user. The base for this recommendation is contained in the grid map of Figure 16, although a final refinement is needed to reach a practical solution. To do so, a reexamination of the actual physical conditions found in the field may be very helpful, especially those occurring when the data were acquired. Figure 17a shows a satellite image of the monitored plot with the irrigation lines (8, 17, and 29) that had a deficit irrigation rate (15% ET_c_) in comparison with the rest (60% ET_c_), as a practical way to induce stress in controlled rows. The experimental plot considered up to Row 32. Note that the high vigor strip already identified in the NDVI maps is slightly noticeable in the satellite image. Figure 17b overlays the position of deficit pipes with the aggregated grid in Figure 16.

**Figure 18:**
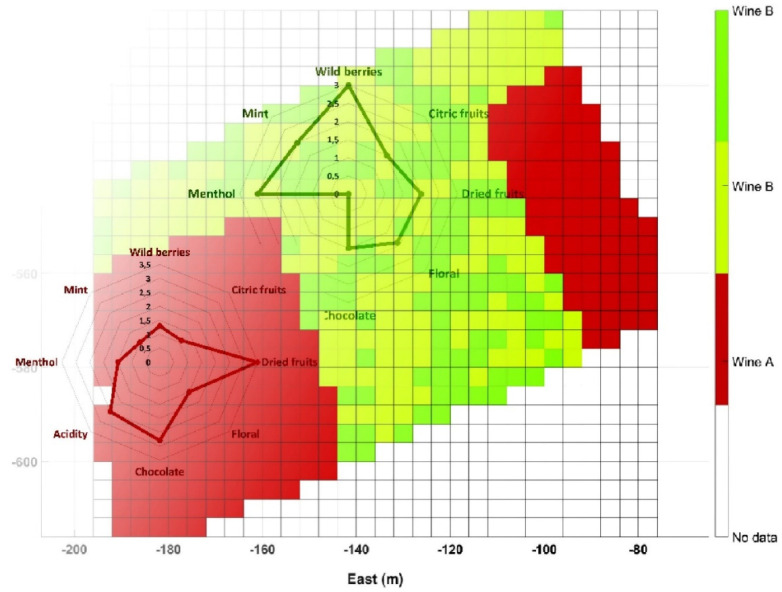
Differential harvesting recommendation map.

The aggregated map in Figure 16 inherits several full rows of high stress from Map B (Figure 13b). According to Figure 17b, these high stress rows approximately replicate the lines under deficit irrigation, which makes sense because the ambient conditions when the map (B) was taken were actually hard, and water deprivation with temperatures of 40 °C and above is prone to create certain stress in plants. The match is not exact, however, because not every row was mapped (traversing alternate rows and measuring only on the right side results in skipping some rows) and the cell size of 4 × 4 somewhat merges some consecutive rows. It would have been more revealing to maintain blocks of at least six adjacent rows with the same irrigation pattern. Nevertheless, in regular production conditions, all rows will get the appropriate water rate, and stress will be determined by other physical properties of the plot such as elevation, orography, and soil, in addition to atmospheric conditions. With these premises, the aggregated map in Figure 16 was simplified to represent two homogeneous zones in which differential harvesting can be applied in practice, as illustrated in Figure 18. These two zones approximately coincide with the wine-making practices followed in this vineyard; microvinification and wine tasting sessions undertaken along the VineScout project revealed distinct properties for the wines coming from the vigor strip zone and from the central-west area, as indicated in Figure 18. The aroma ratings of the two wines shown in the figure below belong to the 2018 harvest that was microvinified in 2019 (the grapes of 2019 season had to be microvinified in 2020, but, due to restrictions derived from the COVID-19 pandemic, this laboratory work was suspended), but it provides end-users with a reasonable expectation for upcoming seasons. These ratings prove that the final traits are distinct for each zone; it will be up to the customers to decide which option better fits their palates (and their pockets). This is, indeed, the final stage of the data-driven recommendation. Now, it is the grower’s turn to make the final decision, based—or not—on the harvesting map in Figure 18. For such decision, other circumstances such as harvesting costs, the availability of a combine harvester versus pickers, and other winery logistics, for example, may exert a significant weight on the ultimate decision.

## 5. Conclusions

An optimized architecture for crop sensing requires choosing, placing, and integrating non-invasive sensors such that field data reliability becomes the highest possible. This is not trivial, and picking the finest alternative among multiple sensing choices and applying reinforcing techniques such as redundancy usually make a difference in performance. The potential of big data has been stated for many disciplines, yet agriculture is not at such state of maturity. Although this is arguable, the reality is that consistent field data taken regularly from the close environment of plants are scarce. This research proposes a methodology to gather massive data—as a forerunner of big data—by combining robotics and proximal sensing. The contribution of this work and the innovative steps undertaken with it include a methodology for autonomous mapping of orchards from ground platforms, the massive sampling of crops with over 20,000 points/ha following a systematic procedure to do so, the high resolution monitoring of NDVI at less than 1 m, the calculation and registration of the precise geographic localization of canopies rather than vehicle trajectories, the tracking of the environment for each plant using global references with a local origin, the way the robotic platform for monitoring operates, and the development of an unsupervised automatic system to create two types of wine (systematic classification for differential harvesting). In addition, massive data were collected over several years and are available for other researchers. 

Our current experience is that average growers believe that an automated machine “just” for collecting data, regardless of their truthfulness and usefulness, does not justify the complexity of the system. Most farmers believe that a robot, or intelligent vehicle in a general formulation of this problem, should also make some “useful” work that pays off the investment. However, the power contained in massive data is not easy to grasp, even with today’s experience with data-driven approaches, which are still young as farming tools. The use case described above ended up making a clear easy-to-execute recommendation, which fell quite close to the end-user practice of previous years in that vineyard. It provided an explanatory example to illustrate the philosophy proposed in this article with only three variables: the spectral index NDVI, the air temperature, and the canopy temperature. However, the robot recorded many other parameters for every point, as the relative humidity, barometric pressure, CO_2_, the photochemical reflectance index, and the reflectance response of both canopy and ambient illumination at wavelengths centered on 650, 810, 570, and 532 nm. Putting all these data at work to yield knowledge value appears to be a titanic effort for an average research team, but data processing analytics and multi-access cloud computing are making exceptional progress to elucidate complex relations and phenomena behind ever-growing amounts of data. When these relations get unleashed and growers actually trust their outcomes, field scouting and proximal monitoring will turn into a need for a sustainable food production in the 21st century.

## Figures and Tables

**Table 1:** Mapping sessions providing data for a use case on harvesting zoning in vineyards.

Map	Date	Initiation Time	Duration (min)	Number of Points	Number of Rows	Area m^2^	DP (Points/m^2^)
A	11 June 2019	15:50	171	20,124	17	5421	3.7
B	22 July 2019	15:34	124	21,367	20	7991	2.7
C	9 September 2020	14:29	71	14,856	14	6475	2.3

## Data Availability

Original data derived from the VineScout research project, from which this paper extracts relevant information and experience, can be accessed in the following address: https://zenodo.org/record/4432057#.X_w94BZ7mXJ.
